# Cost-Effectiveness of Chagas Disease Vector Control Strategies in Northwestern Argentina

**DOI:** 10.1371/journal.pntd.0000363

**Published:** 2009-01-20

**Authors:** Gonzalo M. Vazquez-Prokopec, Cynthia Spillmann, Mario Zaidenberg, Uriel Kitron, Ricardo E. Gürtler

**Affiliations:** 1 Laboratorio de Eco-Epidemiología, Universidad de Buenos Aires, Ciudad Universitaria, Buenos Aires, Argentina; 2 Department of Environmental Studies, Emory University, Atlanta, Georgia, United States of America; 3 Coordinación Nacional de Control de Vectores, Ministerio de Salud de la Nación, Córdoba, Argentina; 4 Coordinación Nacional de Control de Vectores, Delegación Salta, Salta, Argentina; Liverpool School of Tropical Medicine, United Kingdom

## Abstract

**Background:**

Control and prevention of Chagas disease rely mostly on residual spraying of insecticides. In Argentina, vector control shifted from a vertical to a fully horizontal strategy based on community participation between 1992 and 2004. The effects of such strategy on *Triatoma infestans*, the main domestic vector, and on disease transmission have not been assessed.

**Methods and Findings:**

Based on retrospective (1993–2004) records from the Argentinean Ministry of Health for the Moreno Department, Northwestern Argentina, we performed a cost-effectiveness (CE) analysis and compared the observed CE of the fully horizontal vector control strategy with the expected CE for a vertical or a mixed (i.e., vertical attack phase followed by horizontal surveillance) strategy. Total direct costs (in 2004 US$) of the horizontal and mixed strategies were, respectively, 3.3 and 1.7 times lower than the costs of the vertical strategy, due to reductions in personnel costs. The estimated CE ratios for the vertical, mixed and horizontal strategies were US$132, US$82 and US$45 per averted human case, respectively. When per diems were excluded from the costs (i.e., simulating the decentralization of control activities), the CE of vertical, mixed and horizontal strategies was reduced to US$60, US$42 and US$32 per averted case, respectively.

**Conclusions and Significance:**

The mixed strategy would have averted between 1.6 and 4.0 times more human cases than the fully horizontal strategy, and would have been the most cost-effective option to interrupt parasite transmission in the Department. In rural and dispersed areas where waning vertical vector programs cannot accomplish full insecticide coverage, alternative strategies need to be developed. If properly implemented, community participation represents not only the most appealing but also the most cost-effective alternative to accomplish such objectives.

## Author Summary

Despite decreasing rates of prevalence and incidence, Chagas disease remains a serious problem in Latin America, especially for the rural poor. Without vaccines, control and prevention rely mostly on residual spraying of insecticides. Under the aegis of the Southern Cone Initiative, and in agreement with global trends in decentralization of the health systems, in 1992 the Argentinean vector control launched a new vector control program based on community participation. The present study represents the first thorough evaluation of the overall performance of such vector control program and the first comparative assessment of the cost-effectiveness of different vector control strategies in a highly endemic rural area of northwestern Argentina. Supported by results of independent studies, the present work shows that in rural, poor and dispersed areas of the Gran Chaco region, the implementation of a mixed (i.e., vertical attack phase followed by horizontal surveillance) strategy constantly supervised and supported by national or local vector control programs would be the most cost-effective option to interrupt vector-borne transmission of Chagas disease.

## Introduction

Over the past 15 years, the burden of Chagas disease has been significantly reduced (from ∼30 million human cases in 1990 to ∼9–11 million in 2006) as a consequence of the direct actions promoted by several multinational regional initiatives [1],[2]. The key for such success was the long term implementation of residual insecticide applications to kill triatomine bugs, the screening of blood donors for the presence of *Trypanosoma cruzi*, and the treatment of infected infants born to infected mothers [3]. In the Southern Cone, disease transmission by the main vector, *Triatoma infestans*, was interrupted in Uruguay, Chile and Brazil and in southern Argentina [1],[3]. However, limited success was obtained in the Gran Chaco region of northern Argentina, Bolivia and Paraguay (the core of *T. infestans* distribution) where Chagas disease is still highly prevalent.

Within its 1.3 million km^2^, the Gran Chaco provides favorable conditions for the development of Chagas and other neglected diseases, including high levels of poverty and social exclusion, low population density, population mostly rural, subsistence economy, and a weak health system [4],[5]. Recent estimations of Chagas disease prevalence in rural populations of this region show values ranging from 25% to 45% in Argentina, 17% to 49% in Bolivia and 14% to 56% in Paraguay [5],[6],[7], much higher than the overall 1.7% estimated for the Southern Cone countries [2]. Furthermore, the lack of effectiveness of pyrethroid insecticides in peridomestic habitats [8],[9] coupled with the presence of sylvatic populations in Bolivia and Argentina [10] [L.A. Ceballos, unpublished results] and the emergence of insecticide resistance in Argentina and Bolivia [11],[12] renders the elimination of *T. infestans* from the Gran Chaco an elusive challenge.

In Argentina, Chagas disease vector control began in 1962 with the creation of the National Chagas Service (NCS) [13],[14]. Inspired by the old malaria programs, NCS established a vertical and centralized structure based on the application of insecticides (mostly HCH and organophosphates) by qualified personnel. Overall, the program strongly reduced *T. infestans* infestation and *T. cruzi* seroprevalence [14],[15], but failed to achieve full coverage of insecticide applications (as late as 1990, many districts in the Gran Chaco have not yet been sprayed) and to interrupt disease transmission. As a consequence of decentralization and reduced health budgets, by the end of 1980's NCS did not have enough resources to maintain a vertical structure nor to warrant the continuity and contiguity of vector control actions.

Aware of these limitations, NCS started researching on alternative vector control strategies [16],[17]. Based on promising field results [16], and under the aegis of the Southern Cone Initiative, in 1992 NCS launched a new vector control program (“Plan Ramón Carrillo”) based on community participation and on the incorporation of appropriate technology [14],[17],[18]. This new strategy was embedded in the Primary Health Care (PHC) system of Argentina, and included the transference of knowledge and practices of control and surveillance of *T. infestans* to PHC agents, community leaders and rural villagers, who became the first line of *T. infestans* control [14],[17],[19]. During 1993–2001, 15,500 community leaders sprayed with residual insecticides all of the 961,500 houses in the endemic area during the attack phase; 85% of such houses were under community-based vector surveillance [14]. As a consequence of the vector control activities, five provinces, all outside the Gran Chaco, were certified free of vector-borne transmission by 2001 [19]. However, a different scenario was observed in the Argentinean Gran Chaco, with 5 of its 9 provinces reporting vector-borne transmission of Chagas disease by the year 2000 [14]. An evaluation of the effects of the horizontal strategy at the district-wide level in this region is lacking.

In its conception, the horizontal strategy involved the participation of rural communities only in the surveillance phase [16]. However, budget and personnel constraints forced NCS to implement a fully horizontal strategy (i.e., community participation in both the attack and surveillance phases), with the consequent loss of quality of insecticide applications targeting the prevailing high bug infestation levels. Although the horizontal strategy was originally thought to increase the coverage and frequency of insecticide applications while saving the costs of salaries due to the incorporation of unpaid personnel [16],[20],[21], no direct comparative cost-effectiveness (CE) analysis between the horizontal and the preceding vertical strategy was performed to date.

As a part of a larger project on the eco-epidemiology of Chagas disease in northern Argentina, the objectives of the present study were to assess the effects of the horizontal vector control strategy on the prevalence of infestation by *T. infestans* and on the occurrence of human acute cases over a 12-year period (1993–2004) in the Moreno department; and to perform a comparative cost-effectiveness analysis between different vector control strategies (fully horizontal, vertical and mixed) in a highly endemic district of the Argentine Chaco.

## Materials and Methods

### Study area

We analyzed longitudinal data from the NCS for the Moreno Department (centroid at 62° 26′ W, 27° 15′ S), located in the Province of Santiago del Estero, northwestern Argentina (Figure S1). This district was chosen because: a) it is located in the Gran Chaco region; b) historically it presented the highest rates of disease incidence and *T. infestans* infestation; c) all previous control programs failed to reach full coverage of spraying activities; d) an ongoing long-term longitudinal study [22] developed in five rural communities of the Department allowed us to derive key parameters for the present study.

In 2001, Moreno had approximately 25,000 habitants and 5,439 houses, 54% of which were rural houses belonging to 275 communities [23]; most of the rural communities (75%) consisted of 1–10 houses (Figure S1). Health infrastructure in Moreno is composed of three hospitals located in the three major cities, and approximately 22 PHC centers scattered among rural communities. Rural houses usually have adobe walls and thatched roofs, one or two bedrooms, and a 5–10 m wide veranda in the front. The peridomestic environment includes structures that do not share a roof with the bedrooms, such as storerooms, chicken coops and corrals. Exploitation of forest resources (hardwood for charcoal and logs, hunting), raising goats (and cattle) and subsistence agriculture are the main sources of income of rural villagers.

### Study design

Under the horizontal strategy launched in 1992, NCS activities focused on: a) training of local villagers in spraying with pyrethroid insecticides and in bug detection activities; b) spraying of rural communities when a human acute case was detected; c) evaluating domiciles and peridomiciles for the presence of *T. infestans* bugs, and d) the delivery of insecticides, manual compression sprayers, and other supplies to all community leaders. Training workshops for villagers took place at each local school. Workshops provided basic information on Chagas disease epidemiology, and training in insecticide spraying methods and detection of domestic infestation using sensor boxes [18]. At least one resident or PHC agent from every community was selected as a “leader”, and was in charge of storing and distributing the insecticides and sprayers to the villagers who requested them. Each leader was provided with a 5-liter manual compression sprayer, pyrethroid insecticides, and forms to report the spraying activities to NCS personnel on a regular basis [16]. No salary was paid to leaders for their duties.

Insecticide was distributed in small bottles (doses) with the amount of insecticide necessary to fill a 5-liter manual compression sprayer. Villagers were in charge of spraying all domestic and peridomestic structures in their house. After spraying, villagers had to return the manual compression sprayers to the leader, indicating the number of insecticide doses applied, and whether they found *T. infestans* bugs before, during, or immediately after spraying. The monthly number of sprayed houses, the amount of insecticide and domestic boxes used, and the number of house compounds infested by *T. infestans* in domiciles and peridomestic habitats were then reported by leaders to NCS. The program scheme included an attack phase with two spraying rounds of every rural house separated by six months. After the first or second spraying rounds a community was considered under surveillance phase. Suspension concentrate (SC) deltamethrin applied at 25 mg/m^2^ or 20% SC cypermethrin at 125 mg/m^2^ were the insecticides and doses most commonly used (Table S1).

### Cost-effectiveness analysis

We performed a generalized CE analysis [24] and compared the observed CE of the fully horizontal vector control strategy with the expected CE of a vertical or a mixed strategy (i.e., vertical attack phase followed by a horizontal surveillance phase). Generalized CE analysis is based on the evaluation of a suite of interventions against the counterfactual of “doing nothing”, thereby providing a unique framework for evaluating and comparing health interventions, and a gateway for identifying opportunities to improve them.

Direct and indirect costs were estimated separately for the attack and surveillance phases. Direct costs included staff (salaries and per-diems), supplies (consumables used for insecticide spraying and vector surveillance) and mobility (fuel and minor vehicle fixes during fieldwork) (see Text S1 for more details). Straight line depreciation was used to reflect the cost of the use of vehicles (10 years) and manual spraying compressors (5 years). Indirect costs included the maintenance of vehicles and the payment of salaries during the time in which personnel was not assigned to field activities. Costs in Argentine pesos were inflated to 2004 US dollars. Costs were only estimated for activities performed in rural communities. Observed costs for the implementation of the fully horizontal strategy were obtained from NCS records, whereas for the vertical and mixed strategies costs were estimated based on the number of houses of Moreno and the personnel and supplies needed for each strategy (see Text S1 for more details).

The number of Chagas disease human cases (symptomatic and asymptomatic) averted by each strategy was chosen as a measure of their effectiveness. Averted cases were estimated as the difference between the number of human cases observed (horizontal) or expected (vertical and mixed) for each strategy and the number of cases expected in the absence of vector control actions. The number of human cases (*I*) was estimated by applying the following discrete model: *I(t)* = λ_t_*S_t_ ; where λ_t_ represents the instantaneous incidence rate and *S_t_* the number of susceptible individuals in year *t*. Estimation of averted cases was based on the following assumptions: (1) the acquisition of infection is independent of age and sex.; (2) infection is irreversible; (3) mortality, immigration and emigration are negligible; (4) on average, each year there were 631 live births [23]; (5) congenital transmission is negligible; (6) the susceptible population at year 0 is equivalent to 67.7% of total rural population [22]; (7) in the absence of control actions, the instantaneous incidence rate (λ) is constant in time and space and equivalent to the observed value in rural communities of the Moreno Department in 1992 in the absence of control interventions (4.3 per 100 person-years) [25]; (8) reported symptomatic cases are only 7% of total cases [26].

Cost-effectiveness was estimated as the ratio of direct or indirect costs to the number of averted cases, and expressed as 2004 US dollars per averted case. The strategies evaluated were: 1) fully horizontal; 2) vertical (assuming interruption of disease transmission after the attack phase); 3) mixed with vector-borne transmission (i.e., a scenario with persistent transmission throughout the surveillance phase), and 4) mixed without vector-borne transmission (i.e., the attack phase effectively interrupted vector-borne transmission). A sensitivity analysis was performed to evaluate the relative effects of individual key parameters on the absolute value of the CE ratio.

To assess the long term effects of each strategy, CE ratios were projected over a 25-year period considering a 12.8% inflation rate (the 2002–2006 average for Argentina) [27] and a 3% discounting rate per year. For the vertical strategy, an optimistic scenario in which *T. infestans* could be eliminated from Moreno after 10 years of sustained vector control actions was considered. For the 25-year projection, total costs (and hence, CE ratios) accrued in the vertical strategy after year 10 were considered zero due to the interruption of NCS visits after the elimination of *T. infestans*.

### Statistical analysis

Because vector-borne transmission of *T. cruzi* occurs mostly in rural or peri-urban areas, we excluded the three main cities of Moreno (totaling 2,528 houses) from all the analyses. To compare the prevalence of domestic infestation according to the number of times a community was sprayed, we applied Kruskal-Wallis tests with Dunn contrasts [28]. Multiple lineal regression analysis was applied to test whether spraying coverage (i.e., the percentage of houses in the community that were sprayed in the most recent round) and the number of times the community was sprayed from 1993 to 2000 were significantly associated with the prevalence of domestic infestation in year 2000 (the year with more simultaneous records of community infestation). Statistical analyses were performed using SPSS 14.0 (SPSS Inc., Chicago, IL) and STATA 9.1 (Stata Corp, College Station, TX).

## Results

### Evaluation of the fully horizontal control program

Of the 275 rural communities found in Moreno in 1993, 242 (88%) were sprayed with insecticides at least once during 1993–2004 and were thus considered under vector surveillance. The remaining 33 communities were only visited by NCS personnel for community training, insecticide delivery or vector evaluations, but were never registered as sprayed. Most (79%) of these rural communities had an average of 1 to 4 houses. Only 55 (23%) of the rural communities declared under vector surveillance had two insecticide spray cycles under the attack phase.

Villagers performed 79% of the 5,759 insecticide sprays registered in Moreno (Table S1). The total average number of insecticide doses per household was 7.0 during the attack phase and 5.3 during the surveillance phase (Table S1). A total of 1,793 insecticide fumigant canisters were delivered during 1993–2004, at a rate of 146 and 152 canisters per year in the attack and surveillance phases, respectively. Moreover, a total of 12,982 domestic biosensor boxes were delivered to the communities for vector surveillance (Table S1).

**Figure 1:**
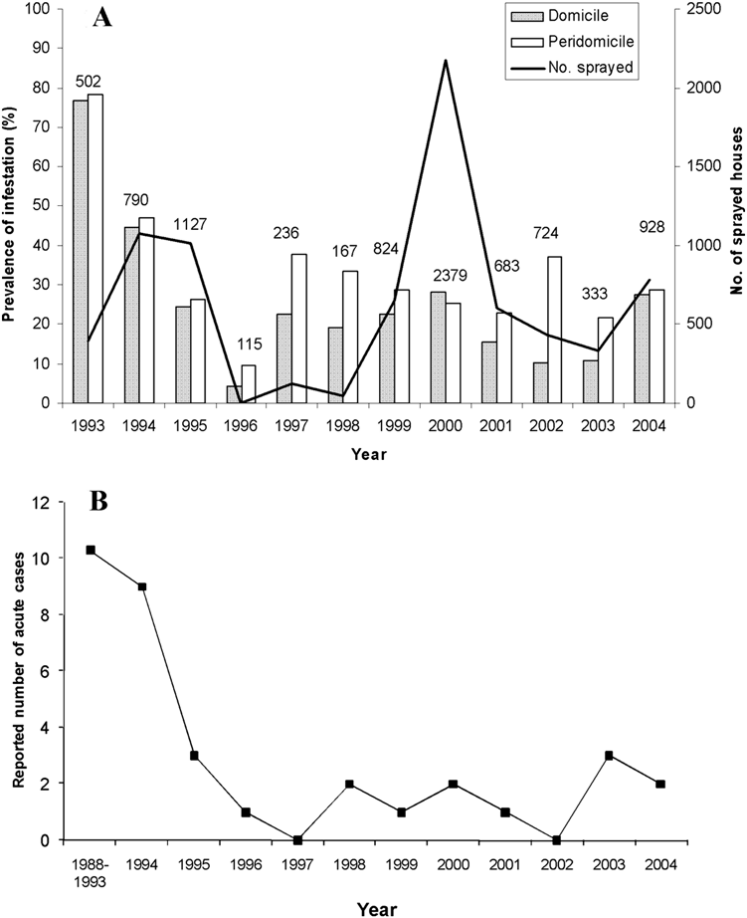
Long-term effects of the fully horizontal strategy. (A) Domestic and peridomestic prevalence of infestation by Triatoma infestans (bars) and number of rural houses sprayed during 1993–2004 (line) in Moreno Department. Numbers on top of bars represent the total number of surveyed houses. (B) Reported number of Chagas disease cases in Moreno Department during 1988–1993 (mean) and 1994–2004.

The prevalence of infestation by *T. infestans* in rural domiciles was 77% in 1993 and decreased to 4% by 1996 (Figure 1A), coinciding with the attack phase. After 1996 domestic infestation fluctuated between 10% and 28%. Peridomestic infestation followed the same trend as domestic infestation, with a decline from 78% to 9% by 1996, and ranging from 22% to 38% during 1997–2004 (Figure 1A). The prevalence of infestation in 25 communities during 1999–2001 was positively correlated with the infestation prevalence assessed by timed manual collections performed by NCS staff in the same communities in 2002 in domiciles (r = 0.45, *P*<0.02), but not in peridomiciles (r = −0.14, *P*>0.4). Because leaders' reports likely underestimated peridomestic infestation (with which they have less contact), this information will not be analyzed hereafter.

The initial attack phase apparently produced a downward trend in the reported number of human acute cases, from an average of 10 per year during 1988–1993 to 0 in 1997 (Figure 1B). From 1998 to 2004 the annual number of cases fluctuated between 0 and 3 with no clear trend. All reported cases were symptomatic, and referred for standard treatment at Hospital Independencia in Santiago del Estero's capital.

**Figure 2:**
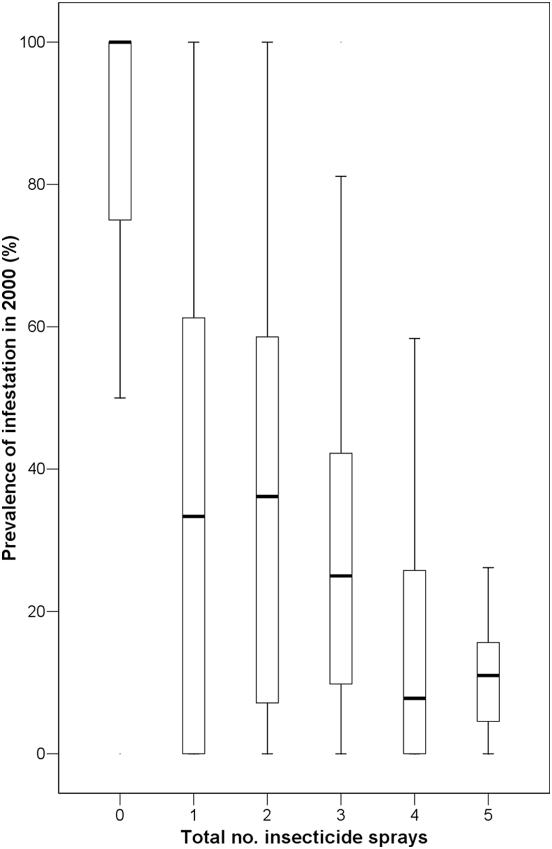
Effects of the frequency of residual insecticide spraying of individual rural communities during 1993–2000 on the prevalence of domestic infestation by *T. infestans* observed in 2000 in Moreno Department. Bold lines represent the median, boxes the first and third quartiles, and vertical lines the range.

Domestic infestation prevalence varied significantly with the number of times each community was reported as sprayed with insecticides by rural villagers (Kruskal-Wallis χ^2^ = 17.9; g.l. = 4; *P* = 0.003) (Figure 2). In communities reported as never sprayed since 1993, the median domestic infestation prevalence was 100% (Figure 2). In communities registered as sprayed once during 1993–2000, a significant reduction in the median domestic infestation prevalence was observed (Dunn contrast, Q = 3.02; g.l. = 1; *P*<0.05). The increase of insecticide spraying frequency from one to three was not followed by a significant reduction in domestic infestation prevalence (Q<2.93; *P*>0.05). However, when communities were reported as sprayed four or more times during 1993–2000, a significant reduction in the median domestic infestation prevalence was observed (Q = 4.08; g.l. = 1; *P*<0.05).

The prevalence of domestic infestation by *T. infestans* in 2000 was significantly and positively associated with the time since last insecticide spray (multiple linear regression coefficient, beta = 0.39, t = 3.34, *P*<0.001) and negatively associated with the coverage of the last insecticide spray (i.e., beta = −0.38, t = −3.28, *P*≤0.001). On average, communities with a domestic infestation prevalence ≥50% in 2000 were sprayed 5.0 (Standard Deviation, SD, 1.8) years earlier, whereas communities with domestic infestation prevalences ≤50% were sprayed 3.0 (SD, 2.0) years earlier. The mean coverage of the last insecticide spray was 79% (SD, 28%) for communities with domestic infestation prevalence ≥50% in 2000, and 84% (SD, 24%) for communities with domestic infestation prevalence ≤50%.

### Cost-effectiveness analysis

**Figure 3:**
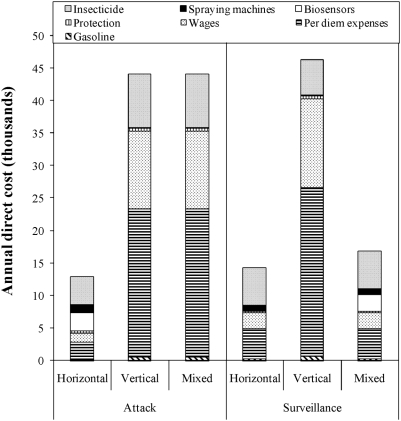
Annual direct costs (in 2004 US$) of the implementation of a fully horizontal (observed data), vertical or mixed (estimated data) vector control strategy in rural communities of Moreno Department.

Total cost (direct and indirect) of the fully horizontal strategy implemented in Moreno during 1993–2004 was $309,426, of which 47% corresponded to indirect costs (Table S2). Indirect costs represented 38% of the total $849,625 estimated for the vertical strategy (Table S3) and 42% of the $582,885 estimated for the mixed strategy (Table S4). Annual direct costs of the horizontal strategy were between 3.4 (attack) and 3.2 (surveillance) times lower than the annual direct costs of the vertical strategy (Figure 3). The cost in personnel (salaries and perdiems) was the cause of the marked difference between strategies. Personnel costs for the vertical strategy were 8.6 (attack) and 5.6 (surveillance) times higher than personnel costs for the horizontal strategy (Figure 3). The total direct cost of spraying a single house during the attack phase was US$ 15 for the horizontal strategy and US$ 38 for the mixed and vertical strategies, whereas the cost of surveying a single house was US$ 17 for the horizontal strategy, US$ 20 for the mixed strategy and US$ 22 for the vertical strategy.

The CE ratio of each strategy (expressed in 2004 US$ per averted case) is presented in Table 1. The lower the coefficient the more cost-effective a strategy (i.e., less money would be needed to avert a single case). Although the fully horizontal strategy showed direct CE ratios 1.9–3.3 times lower than the other strategies, the estimated numbers of human cases were 1.6 to 4.0 times higher than for the remaining strategies (Table 1). When those strategies that may accomplish the interruption of disease transmission (i.e., vertical and mixed WoT) are compared, it can be seen that the strategy Mixed WoT would be the most cost-effective (Table 1).

**Table 1:** Cost-effectiveness (total and direct) associated with the implementation of horizontal, vertical and mixed vector control strategies in rural communities of the Moreno Department, Argentina, during 1993–2004.

	Control strategy			
	Horizontal^1^	Vertical^2^	Mixed WT^3^	Mixed WoT^4^
Total cost*	309,426	849,625	582,885	582,885
Direct cost*	165,101	545,802	339,373	339,373
Total number of cases	580	145	365	145
Averted infections	3,709	4,144	3,924	4,144
Total Cost-effectiveness	83	205	149	141
Direct cost-effectiveness	45	132	86	82

*: In 2004 US$.

1 Observed values for a horizontal strategy based on community participation both in the attack and the surveillance phases of intervention.

2 Estimated values for a vertical strategy with two spraying cycles in the attack phase and a surveillance phase based on active detection of *T. infestans* infestation by NCS staff.

3 Estimated values for a mixed strategy (two spraying rounds in the attack phase performed by NCS staff followed by a surveillance phase based on community participation) in which vector-borne transmission of *T. cruzi* occurs (WT).

4 Estimated values for a mixed strategy that successfully interrupted vector-borne transmission of *T. cruzi* (WoT).

**Figure 4:**
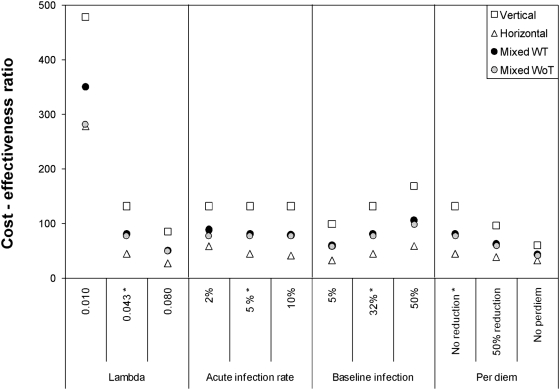
Sensitivity analysis of the different parameters used to estimate the direct cost-effectiveness of each vector control strategy in Moreno Department. *Lambda* refers to disease incidence (cases per year); *acute infection rate* to the percentage of total cases represented by notified acute cases, and *per diem* represents a 50% reduction or the complete elimination of per diems. * indicates the baseline direct CE values. Mixed WT represents a mixed strategy that successfully interrupted disease transmission, whereas mixed WoT a mixed strategy that did not interrupted disease transmission.

Figure 4 shows the results of the sensitivity analysis of CE to various parameters. Changes in the incidence rate (lambda) exerted the highest variation of the direct CE ratio for all strategies. At low incidence rates (0.01 cases per year), horizontal and mixed WoT strategies presented similar and lower CE ratios than the vertical strategy (ΔCE = 201), whereas at high incidence rates (0.08 cases per year) the difference in CE ratios between strategies was less marked (ΔCE = 58 between horizontal and vertical). Variations in the acute infection rate or the baseline human infection prevalence did not affect CE values greatly (range of ΔCE between strategies, 66–109), with the horizontal strategy presenting always lower CE ratios than the mixed and vertical strategies (Figure 4). The last panel of Figure 4 shows the variation of CE ratios due to changes in perdiems (from a 50% reduction to the complete elimination), a scenario compatible with the decentralization of vector control activities. The elimination of perdiem expenses reduced the CE ratio of the vertical and mixed strategies to values closer to the CE ratio of the horizontal strategy (ΔCE between horizontal and vertical strategies = 28).

**Figure 5:**
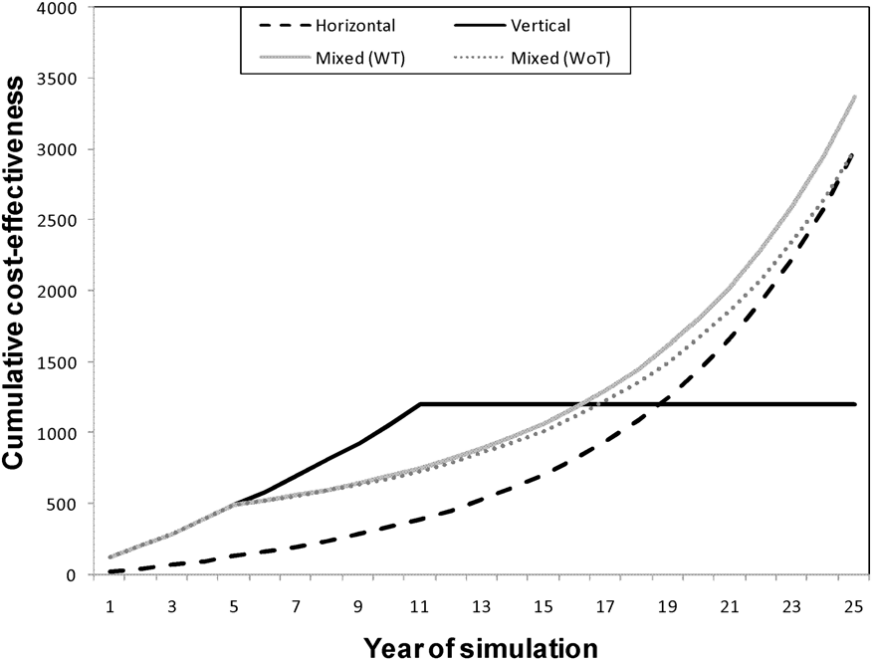
Cumulative projection of the direct cost-effectiveness ratio (expressed in US$ per averted case) for each vector control strategy. Mixed WT represents a mixed strategy that successfully interrupted disease transmission, whereas mixed WoT a mixed strategy that did not interrupted disease transmission.

The long-term effectiveness of each strategy was evaluated by projecting the annual direct CE ratios over a 25-yr period (Figure 5). For the vertical strategy, a scenario that assumed the elimination of *T. infestans* (and the suppression of the costs associated with vector control) after 10 years was evaluated. Figure 5 shows that the mixed and fully horizontal strategies would be more cost-effective than the vertical strategy for up to 16–19 years of interventions, and that the CE of the horizontal and mixed strategies would converge after 21 years of interventions.

## Discussion

As with other vector-borne diseases, the incorporation of community participation in Chagas' disease control and prevention evolved in response to the failure of some vertical programs to achieve their main objectives (originally, vector elimination and interruption of disease transmission) borne, in part, by the acute limitations in personnel and financial support of the health system [18],[20],[21]. The present study represents the first thorough evaluation of the overall performance of a horizontal Chagas' disease vector control program and the first comparative assessment of the CE of different vector control strategies in a highly endemic rural area of Argentina. The results derived from our work may help NCS and other vector control agencies to better plan and design cost-effective control interventions against Chagas' disease vectors.

To achieve significant levels of vector control in endemic areas, Chagas disease control actions need to be sustained over time [22],[29],[30]. In many Latin American countries, the current scenario of partial decentralization of health services, increased poverty, lack of political interest and declining funding for vector control activities represent a serious challenge for the persistence of vertical control strategies [29]. Furthermore, in rural and dispersed areas where waning vertical vector programs cannot accomplish full coverage, alternative strategies need to be developed. The incorporation of participatory approaches against vector borne diseases not only has proven to be cost-effective but also important for the sustainability of control programs [20],[21],[22],[30],[31]. Our analysis shows that the implementation of a mixed strategy would have averted between 1.6 and 4.0 times more human cases than the fully horizontal strategy and, given the realities observed in the ground, would have been the most cost-effective option to interrupt parasite transmission. If properly implemented, community participation represents not only the most appealing but also the most cost-effective alternative to control Chagas disease vectors in resource-constrained settings.

When CE ratio projections were compared, it was clear that the main difference between strategies arises with the potential elimination of *T. infestans*, since vector elimination from a defined region is associated with a significant reduction or even suppression of operational budgets [32]. Although initially it was assumed that 10 years of vector control actions would be enough to accomplish the regional elimination of *T. infestans* from the Southern Cone [33], the eco-epidemiologic reality observed in the Gran Chaco region challenges the feasibility of such assertion [5],[22],[29]. The impossibility of accomplishing the regional elimination of *T. infestans* would have a significant effect in the long-term costs of vector control actions since it would be necessary to maintain a sustained and indefinite surveillance phase to prevent domestic reinfestation by *T. infestans* and interrupt vector-borne transmission of *T. cruzi*.

The success of participatory approaches against tropical diseases is strongly dependent on sustained and continuous collaboration and articulation between external agencies, governments, and communities [30]. In Moreno, such coordination occurred during the attack phase (evidenced by the significant decrease in bug infestation and disease transmission) but not during the surveillance phase. In the latter period, the nearly absence of insecticide sprays and the gradual increase in the prevalence of *T. infestans* infestation were determined by the shortage of insecticide purchases at the central level and by the shift of personnel from the Chagas control program to the recently established dengue control program. Shortage of insecticides, spare parts for compression sprayers and absence of NCS personnel in the field were probably the main obstacles for villagers to continue control activities during the surveillance phase. It is not surprising that new human acute cases of Chagas disease were reported starting in 1998. However, when a mixed control strategy coordinated and supervised by NCS is implemented, *T. infestans* infestation can be significantly reduced and vector-borne transmission of *T. cruzi* successfully interrupted [16],[22],[34],[35],[36].

One of the direct benefits of the inclusion of rural communities in vector control activities is the offset of the high personnel costs associated with vertical, centralized strategies [20],[21]. In Moreno, the implementation of fully horizontal or mixed strategies represented a 1.6–3.5-fold reduction of total direct costs in comparison to a vertical strategy. Such reduction in personnel costs in horizontal strategies, however, came associated with an increase in opportunity costs, because villagers and PHC agents had to divert their available time to control and prevention activities. Given the difficulty to estimate the time villagers devoted to control and surveillance activities, opportunity costs were not included in our cost estimates. Indirect costs represented a significant component of the total cost of each strategy (range, 38–47%), with personnel cost being the most important component. Such high costs were the consequence of NCS centralized structure, since field technicians remained stationed at their central base after long distance travel to the field, devoting their time to activities other than vector control. As shown by the sensitivity analysis, decentralization of NCS structure would be a viable alternative to reduce vector control costs, since perdiem expenses would be sharply reduced and personnel time devoted to vector control, community education and supervision increased.

The measure of effectiveness chosen for the present study allowed the estimation of the cost of averting a single vector-borne Chagas disease human case. However, other measures like the reductions of bug infestation levels, disability adjusted life-years (DALYs) or the quality-adjusted life-years (QALYs) have been proposed for evaluating the effectiveness of control programs [37]. Although vector control actions would have a direct effect on domestic infestation levels by *T. infestans*, such measure of effectiveness was not used in the present study because vector-borne transmission of *T. cruzi* seems to occur at even low bug densities [25],[38]. In addition, DALYs and QALYs were not used because of their known underestimation of the disability weight for chronic parasitic diseases [39],[40], and lack of relevant data (e.g., age-adjusted infection prevalence and mortality rates for each infection phase) to parameterize them.

As most (79%) of the insecticide sprays in Moreno were conducted by villagers, the data herein presented show how effective such sprays were on bug infestation and disease transmission. The prevalence of domestic infestation in communities with an active surveillance and 4 spraying rounds or more during 1993–2000 was 10%, indicating that control actions performed by villagers were sufficient to maintain low infestations but not to eliminate *T. infestans* from domiciles. This may be expected from the observed lower effectiveness of insecticide sprays performed by villagers rather than by NCS technicians, and the resulting higher reinfestation rates. As part of an insecticide trial in 400 houses of Moreno department during 2002–2005, we surveyed heavily infested communities that had been last visited by NCS 4–5 years earlier and found that many villagers did not spray their houses correctly; did not take all the furniture and other items out of the domicile before spraying; changed the dilution of the insecticide to make it last more; and used sprayers in inadequate conditions. Because training workshops had occurred almost 10 years before, most of the young people did not know how to properly spray a house. This demonstrates that community participation cannot be assumed, but has to be systematically supported and promoted [31].

Unit costs of house spraying with pyrethroid insecticides in Moreno were within the cost range estimated for other areas in the Americas, with insecticide costs being a variable (albeit important) budget component of the vector control programs (Table 2). Such costs were much lower and therefore more affordable than the 200–2,000 US$ range estimated for housing improvements [41]. The dependence on residual insecticides for the suppression of disease transmission represents an additional burden for Chagas disease vector control programs because insecticide purchases are negotiated at international market prices. The integration of disease programs (i.e., Chagas and malaria where both diseases overlap) and, particularly, the international call for a significant reduction in insecticide prices allocated for vector-borne disease prevention in developing countries represent, in our opinion, some of the integrated, inter-programmatic, inter-sectoral actions [42] needed for reducing the burden of Chagas disease in the Americas.

**Table 2:** Published primary sources of unit cost per house of controlling triatomine bugs.

Location	Strategy^1^	Cost^2^		% cost of insecticides		Year of survey	Ref
		Attack	Surveillance	Attack	Surveillance		
Dept. Moreno, Santiago del Estero, Argentina.	H	15	17	25	33	1993–2004	This study
	M	38	20	16	10		
	V	38	22	16	25		
Dept. Anta, Province of Salta, Argentina	V	63	2	23	ND	1983–1984	[26]
Dept. Rio Hondo, Province of Santiago del Estero, Argentina	V	60	17	ND	ND	1985	[43]
	H	ND	3	ND	ND		
Municipality of Posse, Goyas State, Brazil.	V	37	8.5	64	ND	1988	[44]
Sud Chichas Province, Bolivia.	V	49	ND	71	ND	1994	[45]
Paraguay	V	29	ND	ND	ND	1988–1991	[46]
Nicaragua	V	5	1	ND	ND	1997	[47]
Colombia	V	48	ND	39	ND	2001	[48]

1 V (vertical), H (fully horizontal), M (mixed).

2 Expressed in US$ the respective survey year.

## Supporting Information

Figure S1 Map of the distribution of villages and number of houses per village in Moreno Department, Province of Santiago del Estero, Argentina
(0.14 MB PDF)

Table S1 Total number of households sprayed, percentage of insecticide sprays performed by villagers and amount of insecticide and domestic biosensors delivered to the community leaders in the Moreno Department in 1993–2004
(0.04 MB PDF)

Table S2 Observed cost (in 2004 US$) of each one of the direct and indirect components related to the implementation of the *T. infestans* fully horizontal control strategy implemented in Moreno Department during 1993–2004
(0.03 MB PDF)

Table S3 Estimated cost (in 2004 US$) of each one of the direct and indirect components related to the implementation of a *T. infestans* vertical control strategy in Moreno Department during 1993–2004
(0.03 MB PDF)

Table S4 Estimated cost (in 2004 US$) of each one of the direct and indirect components related to the implementation of a *T. infestans* mixed (i.e., vertical attack phase followed by horizontal surveillance) strategy in the Moreno Department during 1993–2004
(0.03 MB PDF)

Text S1 Supplementary methods
(0.01 MB PDF)

Alternative Language Abstract S1 Translation of the abstract into Spanish by Gonzalo M. Vazquez-Prokopec
(0.01 MB PDF)
