# Supplementary material for: Cost-Effectiveness of Chagas Disease Vector Control Strategies in Northwestern Argentina
Source: PLoS Negl Trop Dis. 2009 Jan 20;3(1):e363. doi: 10.1371/journal.pntd.0000363 (PMC2613538; doi:10.1371/journal.pntd.0000363)

Figure S1. Map of the distribution of villages and number of houses per village in Moreno Department, Province of Santiago del Estero, Argentina. Inset shows the location of Moreno in Argentina.

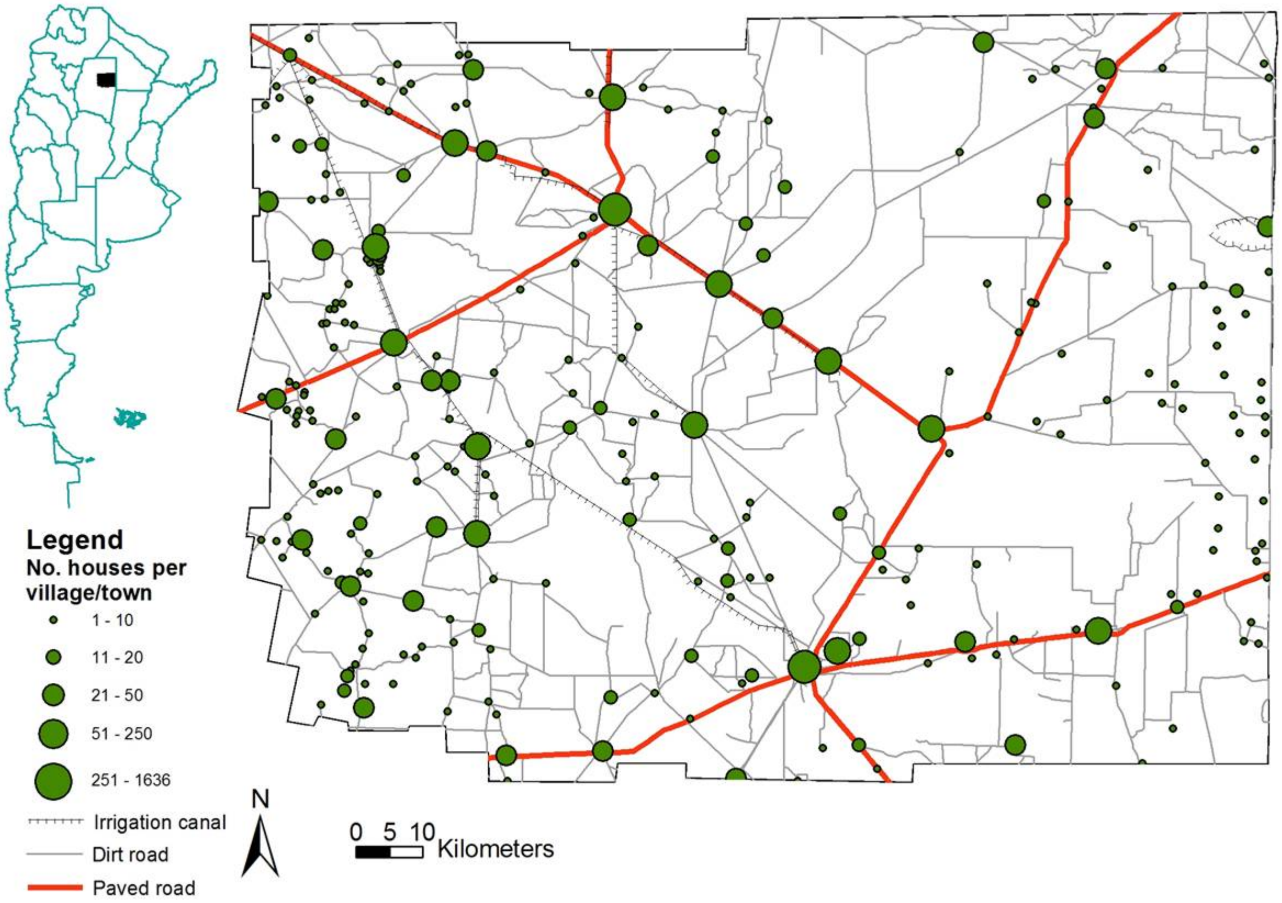

Supplement: Figure S1 — Map of the distribution of villages and number of houses per village in Moreno Department, Province of Santiago del Estero, Argentina (0.14 MB PDF) [file pntd.0000363.s001.pdf]
