# Supplementary material for: Cost-Effectiveness of Chagas Disease Vector Control Strategies in Northwestern Argentina
Source: PLoS Negl Trop Dis. 2009 Jan 20;3(1):e363. doi: 10.1371/journal.pntd.0000363 (PMC2613538; doi:10.1371/journal.pntd.0000363)
Supplement: Table S1 — Total number of households sprayed, percentage of insecticide sprays performed by villagers and amount of insecticide and domestic biosensors delivered to the community leaders in the Moreno Department in 1993–2004 (0.04 MB PDF) [file pntd.0000363.s002.pdf]

Table S1. Total number of households sprayed, percentage of insecticide sprays performed by villagers and amount of insecticide and domestic biosensors delivered to the community leaders in the Moreno Department in 1993-2004.

				Insecticide type (doses[1])							
Control phase	Year	No. houses sprayed (urban + rural)	Houses sprayed by villagers (%)	Delta-methrin (25 mg ai[2]/m^2)	Cipermethrin (125 mg ai/m^2)	Beta-cipermethrin (50 mg ai/m^2)	Alfa-cipermethrin (60 mg ai/m^2)	Lambda-cyhalothrin (33 mg ia/m^2)	Total doses	Fumigant canisters (units)	Domestic biosensors (units)
Attack	1993	395	100	1,010	0	0	0	0	1,010	60	0
	1994	1,762	83	5,164	1,841	0	0	3,103	10,108	411	0
	1995	1,654	59	7,680	2,801	0	0	800	11,281	251	8,723
	1996	105	100	4,037	325	326	0	814	5,502	0	3,139
	1997	327	100	786	98	214	0	521	1,619	10	1,120
	**Subtotal**	**4.243**	**77**	**18,677**	**5,065**	**540**	**0**	**5,238**	**29,520**	**732**	**12,982**
Surveillance	1998	119	71	0	0	26	291	365	682	0	0
	1999	767	96	404	0	2,509	432	1,719	5,064	1	0
	2000	2,476	94	11,422	0	873	1,751	698	14,744	0	0
	2001	723	86	3,116	0	824	154	65	4,159	503	0
	2002	445	74	1,442	0	0	878	0	2,320	453	0
	2003	372	80	520	0	0	733	0	1,253	94	0
	2004	857	32	2,516	0	0	0	0	2,516	10	0
	**Subtotal**	**5.759**	**76**	**19,420**	**0**	**4,232**	**4,239**	**2,847**	**30,738**	**1,061**	**0**
Total		10,002	79	38,097	5,065	4,772	4,239	8,085	60,258	1,793	12,982

[1]Dosis, bottles with the amount of insecticide necessary for charging a 5 L manual compression sprayer. The amounts of insecticide per dosis are: 100 ml for deltamethrin (Agrevo), betacipermethrin (Chemotecnica), alfacipermethrin (Basf), and cypermethrin (Chemotecnica); 3.75 gr for lambdacyhalothrin (Zeneca).
[2]ai, active ingredient.
