# Supplementary material for: Cost-Effectiveness of Chagas Disease Vector Control Strategies in Northwestern Argentina
Source: PLoS Negl Trop Dis. 2009 Jan 20;3(1):e363. doi: 10.1371/journal.pntd.0000363 (PMC2613538; doi:10.1371/journal.pntd.0000363)
Supplement: Table S2 — Observed cost (in 2004 US$) of each one of the direct and indirect components related to the implementation of the T. infestans fully horizontal control strategy implemented in Moreno Department during 1993–2004 (0.03 MB PDF) [file pntd.0000363.s003.pdf]

Table S2. Observed cost (in 2004 US$) of each one of the direct and indirect components related to the implementation of the *T. infestans* fully horizontal control strategy implemented in Moreno Department during 1993-2004.

Cost	Category	Expenses	Attack (1993-1997)	Surveillance (1998-2004)	Total (1993-2004)
Direct	Consumables	Insecticide	21,358	40,630	61,989
		Spraying machines	6,478	6,478	12,956
		Biosensors	13,596	0	13,596
		Protection[1]	1,684	1,684	3,369
	Personnel	Wages	6,983	17,021	24,004
		Per diem	13,239	32,266	45,505
	Mobility	Gasoline	1,006	2,341	3,347
		Vehicle fixes	101	234	335
	Subtotal		*64,446*	*100,655*	*165,101*
Indirect	Personnel	Personnel maintenance[2]	33,997	97,724	131,721
	Mobility	Vehicle maintenance[3]	1,372	11,232	12,605
	Subtotal		*35,369*	*108,956*	*144,326*
	**Total**		**99,815**	**209,612**	**309,427**

[1] Personal protection elements: masks, globes, helmet, goggles.
[2] Represents the cost (in wages) of maintaining the personnel during the time it is not in the field.
[3] Represents the cost in parts, mechanical services and general fixes of the vehicles while not in the field.
