# Supplementary material for: Cost-Effectiveness of Chagas Disease Vector Control Strategies in Northwestern Argentina
Source: PLoS Negl Trop Dis. 2009 Jan 20;3(1):e363. doi: 10.1371/journal.pntd.0000363 (PMC2613538; doi:10.1371/journal.pntd.0000363)
Supplement: Table S3 — Estimated cost (in 2004 US$) of each one of the direct and indirect components related to the implementation of a T. infestans vertical control strategy in Moreno Department during 1993–2004 (0.03 MB PDF) [file pntd.0000363.s004.pdf]

Table S3. Estimated cost (in 2004 US$) of each one of the direct and indirect components related to the implementation of a *T. infestans* vertical control strategy in Moreno Department during 1993-2004.

Cost	Category	Expenses	Attack (1993-1997)	Surveillance (1998-2004)	Total (1993-2004)
Direct	Consumables	Insecticide [1]	41,305	37,542[2]	78,846
		Spraying machines	593	593	1,186
		Biosensors	0	0	0
		Protection [3]	2,752	3,852	6,604
	Personnel	Wages [4]	59,701	95,522	155,223
		Per diem [4]	113,975	182,359	296,334
	Mobility	Gasoline	2,660	4,257	6,917
		Vehicle fixes	266	426	692
	Subtotal		*221,252*	*324,550*	*545,802*
Indirect	Personnel	Personnel maintenance [5]	130,439	160,842	291,281
	Mobility	Vehicle maintenance [6]	4,118	8,425	12,543
	Subtotal		*134,557*	*169,267*	*303,823*
	**Total**		**355,808**	**493,817**	**849,625**

[1] Considering 2 spraying cycles of the 2,911 rural houses of the department in the attack phase, and of 10% of the houses in the surveillance phase.
[2] Estimated from the cost of tetramethrin for timed-manual collections and the cost of deltamethrin for the spraying of infested households.
[3] Personal protection elements: work clothes, masks, globes, helmet, and goggles.
[4] Assuming the activity of 3 groups of 3 NCS technicians working for 35 (attack) or 56 (surveillance) trips per year, each lasting for 17 days.
[5] Represents the cost (in wages) of maintaining the personnel during the time they are not in the field.
[6] Represents the cost in parts, mechanical services and general fixes of the vehicles while not in the field.
