# Supplementary material for: Cost-Effectiveness of Chagas Disease Vector Control Strategies in Northwestern Argentina
Source: PLoS Negl Trop Dis. 2009 Jan 20;3(1):e363. doi: 10.1371/journal.pntd.0000363 (PMC2613538; doi:10.1371/journal.pntd.0000363)
Supplement: Table S4 — Estimated cost (in 2004 US$) of each one of the direct and indirect components related to the implementation of a T. infestans mixed (i.e., vertical attack phase followed by horizontal surveillance) strategy in the Moreno Department during 1993–2004 (0.03 MB PDF) [file pntd.0000363.s005.pdf]

**Table S4.** Estimated cost (in 2004 US$) of each one of the direct and indirect components related to the implementation of a *T. infestans* mixed (i.e., vertical attack phase followed by horizontal surveillance) strategy in the Moreno Department during 1993-2004.

| Cost | Category | Expenses | Attack (1993-1997) | Surveillance (1998-2004) | Total (1993-2004) |
|---|---|---|---|---|---|
| Direct | Consumables | Insecticide [1] | 41,305 | 40,630 | 81,935 |
| | | Spraying machines | 593 | 6,478 | 7,071 |
| | | Biosensors | 0 | 17,466 | 17,466 |
| | | Protection [2] | 2,752 | 1,684 | 4,436 |
| | Personnel | Wages [3] | 59,701 | 17,021 | 76,722 |
| | | Per diem [3] | 113,975 | 32,266 | 146,241 |
| | Mobility | Gasoline | 2,660 | 2,341 | 5,001 |
| | | Vehicle fixes | 266 | 234 | 500 |
| | *Subtotal* | | *221,252* | *118,121* | *339,373* |
| Indirect | Personnel | Personnel maintenance [4] | 130,439 | 97,724 | 228,163 |
| | Mobility | Vehicle maintenance [5] | 4,118 | 11,232 | 15,350 |
| | *Subtotal* | | *134,557* | *108,956* | *243,513* |
| | **Total** | | **355,808** | **227,077** | **582,885** |

[1] Considering 2 spraying cycles of the 2,911 rural houses of the department in the attack phase, and the supply of insecticides to community leaders in the surveillance phase.

[2] Personal protection elements: work clothes, masks, globes, helmet, and goggles.

[3] Assuming the activity of 3 groups of 3 NCS technicians working for 35 (attack) or 56 (surveillance) trips per year, each lasting for 17 days.

[4] Represents the cost (in wages) of maintaining the personnel during the time it is not in the field.

[5] Represents the cost in parts, mechanical services and general fixes of the vehicles while not in the field.
