# Supplementary material for: Cost-Effectiveness of Chagas Disease Vector Control Strategies in Northwestern Argentina
Source: PLoS Negl Trop Dis. 2009 Jan 20;3(1):e363. doi: 10.1371/journal.pntd.0000363 (PMC2613538; doi:10.1371/journal.pntd.0000363)
Supplement: Text S1 — Supplementary methods (0.01 MB PDF) [file pntd.0000363.s006.pdf]

PLoS Neglected Tropical Diseases.

Cost-effectiveness of Chagas Disease Vector Control Strategies in Northwestern Argentina

Gonzalo M. Vazquez-Prokopec, Cynthia Spillmann, Mario Zaidenberg, Uriel Kitron, Ricardo E. Gürtler

Supplementary Methods

Cost estimations. In the fully horizontal strategy, NCS field technicians were organized in teams of two persons per vehicle. The lack of personnel forced NCS to assign only one team to Moreno Department for the attack phase (1993-1997) and an average of 4 (range, 1-6) teams for the surveillance phase (1997-2004). NCS teams traveled to the Moreno Department (from its bases located in Tucuman or Cordoba Provinces, some 500-600 km away) in trips lasting 17-20 days. Each trip consisted of training of new communities, visits to already trained communities for supervision and delivery of supplies (insecticides, forms, and domestic biosensors), and residual spraying of communities where an acute human case was notified.

During the attack phase, a total of 622 person-days was worked by NCS technicians in Moreno, whereas in the surveillance phase the number of days worked increased to 1,516 person-days. Each worker's wage was estimated as the average for 1993-2002 (US$ 337 including the annual bonus or "aguinaldo"). Direct costs were estimated as the number of person-days spent in the field, times US$337/30. On the other hand, the number of person-days not spent in the field (excluding vacation days) was considered as indirect costs of the strategy. Perdiem costs were constant throughout 1993-2004, and equivalent to US$ 22 per day.

Total gas cost was US$ 1,006 during the attack phase and US$ 2,341 during the surveillance phase. Insecticide costs were estimated as the total number of doses of each insecticide type delivered to the community leaders, at US$ 3 per dose. A total of 18,715 doses was estimated to be delivered in rural communities during the attack phase, whereas 28,382 doses were estimated to be delivered during the surveillance phase. Domestic biosensors were delivered at a rate of 4 per house, at a cost of US$ 1.1 per biosensor.

Manual compression spraying machines were delivered at a rate of 1 per trained leader. In communities with more than 30 houses, more than one leader was trained and therefore more than one spraying machine was delivered. A total of 295 spraying machines was distributed during the attack phase. Defective machines were replaced by NCS technicians once during the surveillance phase. The average cost of each spraying

machine for 1993-2004 was US$ 22 (range, US$ 16.9-29.9). With each machine, each leader received the following protection elements: two mouth filters, one pair of security goggles, one disposable jacket, and one pair of rubber gloves. The cost of the protection kit per leader was US$ 5.7. Minor consumable costs incurred in the field such as tire patches, oil and brake fluid refills were estimated by NCS as 10% of the cost of gasoline. Given that all the vehicles used in Moreno were acquired in early 1980, it was assumed that they were completely depreciated at the beginning of the strategy and therefore only vehicle maintenance and parts were considered as vehicle costs.

For the vertical strategy, costs were estimated assuming the spraying and surveillance of all rural houses in Moreno. Given the high reinfestation rates found in the Gran Chaco, it was assumed that a vertical strategy with an attack phase with two spraying rounds and an intensive surveillance phase would interrupt disease transmission and drive *T. infestans* numbers to levels close to elimination. Given the limitations in personnel it was assumed that only 3 teams of three NCS technicians were in charge of spraying and surveillance activities of all 2,911rural houses of Moreno. From our experience in the Moreno Department [1,2], one profesional team sprays an average of 4 rural houses (domicile + peridomicile) a day, or 60 houses on each 17-day trip (assuming 15 effective working days). From these values we estimated that it would take 32 trips (or 4.6 years assuming 7 trips per year) to the three NCS teams to complete the two spraying rounds of all Moreno houses in the attack phase. For the surveillance phase, each team had to evaluate each house (domicile and peridomicile) by timed manual collections with an irritant spray [2] and selectively spray the houses found infested by *T. infestans*. From our experience in Moreno, one team of three NCS technicians evaluates an average of 12 houses a day, or 180 houses per 17-day trip. This means that it will require 5.4 trips to the three teams to evaluate the 2,911 rural houses of Moreno. Moreover, considering that about 291 houses will need to be selectively sprayed each year (see below), and assuming that one team sprays 4 houses a day, each year the surveillance phase would need to be extended for 24 additional days (or 1.6 trips). From these values, we estimated that it would take 7 trips per year to the three NCS teams to survey (evaluate and selectively spray the infested houses) all Moreno houses in the surveillance phase. A total of 56 surveillance trips was estimated to have been performed during 1997-2004, with a total of 150 l of gasoline per trip.

Insecticide estimates for the vertical attack phase were based on the total number of rural houses (2,911) to be sprayed, the amount of insecticide per house (7 doses to spray domestic and peridomestic habitats) and the cost per dose (US$ 3). For the surveillance phase deltamethrin costs were estimated for spraying of residual foci and 0.2% tetramethrin costs for timed manual collections. Selective spraying of reinfested houses was considered as the control measure for the surveillance phase. Information from different field studies performed in the Gran Chaco showed an average reinfestation rate (domestic or peridomestic) of rural houses of 10.5% (range during the 4 years post spraying, 2,9-16,9%) [1,2,3]. Considering such reinfestation rate, the cost for spraying 291 houses a year was estimated (assuming the application of 7 doses per house). Manual compression sprayers for each of the 3 team members were renewed every 2 years at a cost of US$ 22 per machine. For timed manual collections the cost of the irritant spray was estimated assuming that one NCS technician uses one tetramethrin spray approximately every 15 houses, at a cost of US$ 1.2 per spray. For the vertical

strategy, every year each NCS technician would have been provided with protection kits (i.e., mouth respirators, work clothes, boots, gloves, goggles, helmet and protection coat) with a unit cost of US$ 61 per kit.

For the mixed strategy, cost estimates of the vertical attack phase and the horizontal surveillance phase were used. Additionally, for the surveillance phase the cost of domestic biosensors for the 2,911 rural houses (at a rate of 4 per house) and manual compression sprayers for community leaders was estimated.

Supplementary References

1.Cecere MC, Vazquez-Prokopec GM, Ceballos LA, Gurevitz JM, Zárate JE, Zaidenberg M, Kitron U, Gürtler RE. 2006a. Comparative trial of the effectiveness of pyrethroid insecticides against peridomestic populations of *Triatoma infestans* in northwestern Argentina. *J. Med. Entomol.*, 43: 902-909.

2.Gürtler RE, Cecere MC, Canale D, Castañera MB, Chuit R, Cohen JE. 1999. Monitoring house reinfestation by vectors of Chagas disease: a comparative trial of detection methods during a four-year follow-up. *Acta. Trop.*, 72: 213-234.

3.Gürtler RE, Canale DM, Spillmann C, Stariolo R, Salomon OD, Blanco S, Segura EL. 2004. Effectiveness of residual spraying of peridomestic ecotopes with deltamethrin and permethrin on *Triatoma infestans* in rural western Argentina: a district-wide randomized trial. *Bull. World Health Organ.*, 82: 196-205.
