# Supplementary material for: Cost-Effectiveness of Chagas Disease Vector Control Strategies in Northwestern Argentina
Source: PLoS Negl Trop Dis. 2009 Jan 20;3(1):e363. doi: 10.1371/journal.pntd.0000363 (PMC2613538; doi:10.1371/journal.pntd.0000363)
Supplement: Alternative Language Abstract S1 — Translation of the abstract into Spanish by Gonzalo M. Vazquez-Prokopec (0.01 MB PDF) [file pntd.0000363.s007.pdf]

PLoS Neglected Tropical Diseases.

Cost-effectiveness of Chagas Disease Vector Control Strategies in Northwestern Argentina

Gonzalo M. Vazquez-Prokopec, Cynthia Spillmann, Mario Zaidenberg, Uriel Kitron, Ricardo E. Gürtler

Supplementary text.

Translated abstract

Antecedentes. La estrategia más eficaz para prevenir la transmisión vectorial de la enfermedad de Chagas consiste en el rociado de insecticidas con poder residual. A partir del año 1992, el programa de control vectorial de la enfermedad de Chagas de Argentina cambió de una estructura vertical a una horizontal basada en la participación comunitaria. Sin embargo, los efectos de dicho programa sobre la infestación por *Triatoma infestans* y la transmisión vectorial de *Trypanosoma cruzi* no han sido determinados aún.

Métodos y hallazgos. A partir de datos retrospectivos (1992-2004) del Departamento Moreno (Noroeste Argentino) provistos por el Ministerio de Salud de Argentina hemos realizado un análisis de costo-efectividad (CE) comparando la CE observada para la estrategia de control horizontal con la CE esperada para una estrategia vertical y para una estrategia mixta (i.e., fase de ataque vertical seguida por una fase de vigilancia con participación comunitaria). El costo directo total (expresado en US$ del 2004) de las estrategias horizontal y mixta fue, respectivamente, 3,3 y 1,7 veces menor que el costo estimado para la estrategia vertical, debido a la reducción en los costos de personal. Los valores del coeficiente de CE para las estrategias vertical, mixta y horizontal fueron, respectivamente, 132, 82 y 45 US$ por caso humano vectorial evitado. Cuando los viáticos fueron excluidos de los análisis (i.e., simulando la descentralización de las actividades de control) la CE de las estrategias vertical, mixta y horizontal se redujo, respectivamente, a 60, 42 y 32 US$ por caso humano vectorial evitado.

Conclusiones y significancia. La estrategia mixta habría evitado entre 1,6 y 4,0 más casos humanos vectoriales que la estrategias horizontal y habría sido la estrategia más costo-efectiva para interrumpir la transmisión vectorial de *T. cruzi* en Moreno. En áreas rurales y dispersas, donde los escasos recursos de los programas de control no pueden garantizar la cobertura total del rociado de insecticidas, resulta necesario el desarrollo de estrategias de control alternativas. De ser correctamente implementada, la participación comunitaria representaría no solo la alternativa más atractiva pero también la más costo-efectiva para lograr dichos objetivos.
